# Dexamethasone Modulates Nonvisual Opsins, Glucocorticoid Receptor, and Clock Genes in* Danio rerio* ZEM-2S Cells

**DOI:** 10.1155/2017/8459385

**Published:** 2017-05-14

**Authors:** Jennifer Caroline Sousa, Keila Karoline Magalhães-Marques, Sanseray da Silveira Cruz-Machado, Maria Nathalia Moraes, Ana Maria de Lauro Castrucci

**Affiliations:** Department of Physiology, Institute of Biosciences, University of Sao Paulo, Sao Paulo, SP, Brazil

## Abstract

Here we report, for the first time, the differential cellular distribution of two melanopsins (Opn4m1 and Opn4m2) and the effects of GR agonist, dexamethasone, on the expression of these opsins and clock genes, in the photosensitive* D. rerio* ZEM-2S embryonic cells. Immunopositive labeling for Opn4m1 was detected in the cell membrane whereas Opn4m2 labeling shows nuclear localization, which did not change in response to light.* opn4m1*,* opn4m2*,* gr*,* per1b, *and* cry1b *presented an oscillatory profile of expression in LD condition. In both DD and LD condition, dexamethasone (DEX) treatment shifted the peak expression of* per1b *and* cry1b* transcripts to ZT16, which corresponds to the highest* opn4m1 *expression. Interestingly, DEX promoted an increase of* per1b* expression when applied in LD condition but a decrease when the cells were kept under DD condition. Although DEX effects are divergent with different light conditions, the response resulted in clock synchronization in all cases. Taken together, these data demonstrate that* D. rerio* ZEM-2S cells possess a photosensitive system due to melanopsin expression which results in an oscillatory profile of clock genes in response to LD cycle. Moreover, we provide evidence that glucocorticoid acts as a circadian regulator of* D. rerio* peripheral clocks.

## 1. Introduction

Time is a fundamental variable for the manifestation of biological phenomena, and as such it has been systematically investigated since the pioneer chronobiology studies by Halberg [1]. The generation and maintenance of biological rhythms are explained based on a molecular machinery composed of positive and negative feedback loops of transcription and translation of core genes [2–9]. Although major work has been done with rodents, nonmammalian vertebrates have been explored by our group to favor an evolutionary perspective of the fundamentals of the biological clocks [10–17].

*Danio rerio*, the popular* zebrafish*, has been employed as an alternative model to study clock molecular machinery, due to the similarities with the mammalian clock regarding the genes and their feedback loops [18, 19]. On the other hand, unlike mammals, the existence of photosensitive biological clocks in many peripheral organs [11, 20] allows the investigation of processes subjacent to light transduction in peripheral clocks.

The photopigments responsible for converting light into temporal information are assembled in a family of nonimage forming opsins named melanopsins, which were firstly discovered in* Xenopus laevis *melanophores [21], with subsequent description of orthologues in the retina of all vertebrate classes [22–25], and extraocular tissues of some species [26–28]. Data on genomic sequence similarity, chromosomal localization, and phylogeny revealed that melanopsin genes have evolved into two separate groups:* Opn4m*, orthologue of mammalian melanopsin, and* Opn4x*, orthologue of the melanopsin initially cloned from* X. laevis *[24, 25]. Both melanopsins are expressed in fish, amphibians, and birds, while mammals have apparently lost the ancestral* Opn4x *locus during chromosomal reorganization [25]. Surprisingly, in adult* Danio rerio *retina, five melanopsin genes,* opn4m1, opn4m2, opn4m3*,* opn4x1, *and* opn4x2 *[29], are found, although* opn4m1 *and* opn4m2 *are the mostly abundant melanopsins expressed in zebrafish embryonic cells (ZEM-2S) [14]. The high number of melanopsin genes is most probably due to teleost-specific whole genome duplication event which occurred in an ancient fish 350 million years ago [30].

Mammalian melanopsin is expressed by a small subset of retinal ganglion cells and translates light stimulus into neural information, which reaches the suprachiasmatic nucleus (SCN) through the retinohypothalamic tract [31, 32]. By contrast, the wide melanopsin expression in zebrafish peripheral tissues allows this fish to be directly entrained by light/dark cycles even in cultured cells [11, 14, 20, 33–35].

The molecular mechanisms of zebrafish circadian rhythm are similar to the well-known mammalian system and rely on core set of genes composed of* clock*,* bmal*,* per,* and* cry*. Clock:Bmal dimer induces rhythmic expression of* per* and* cry*, while Per and Cry proteins repress their own expression by interacting with Clock:Bmal via the processes of blocking and displacement repressions [36]. In addition, there exists a stabilizing loop, which stimulates or represses* bmal *expression through the clock-controlled genes,* Ror *and* Rev-erb*, respectively. The completion of this feedback system takes about 24 h, thus generating a pattern of circadian rhythm [19].

However, some differences as the multiple clock genes and the light sensitivity make zebrafish a peculiar vertebrate model. Four* period (per1a*,* per1b*,* per2*, and* per3)*, 6* cryptochrome (cry1a*,* cry1b*,* cry2a*,* cry2b*,* cry3*, and* cry4)*, 3* clock (clock1a*,* clock1b,* and* clock2),* and 3* bmal (bmal1a*,* bmal1b,* and* bmal2)* genes have been cloned from* D. rerio *[18, 19, 37, 38]. These multiple copies probably perform specialized functions in the clock regulation: all Cry proteins, except Cry3 and Cry4, and Per proteins inhibit Clock:Bmal [37]. The zebrafish light sensitivity is related to light induction of* per2* and* cry1a* genes via D box-binding factor TEF* (Thyrotroph Embryonic Factor)* [39–41], a signaling pathway which has recently been receiving the researchers' attention [14, 19].

The synchronization between central and peripheral clocks raised the hypothesis that humoral factors are responsible for their communication. Hormones such as glucocorticoids (GCs) became strong candidates as regulatory agents of clock genes, as they are rhythmically produced and released in all vertebrates [42–44]. One-hour pulse of dexamethasone (DEX), a synthetic glucocorticoid analogue, induces the circadian expression of* Per1 *in rat fibroblasts [45]. In fact, mammalian* Per1 *gene possesses the glucocorticoid responsive element (GRE), indicating that GC-receptor complex may directly modulate* Per1 *expression [46]. Mouse primary culture of mesenchymal cells responds with oscillatory transcription of* Per1*,* Per2*,* Per3*,* Cry1*,* Cry2*,* Npas2 *(*Clock *paralogue), and* Bmal1 *and of the clock-controlled genes,* Rev-erb α*/*β* and* Dbp *to GCs [47]. In rat hepatocyte primary culture, GCs also inhibit* Rev-erbα*transcription [48].

Although the literature strongly indicates regulation of mammalian biological clocks by GCs, the mechanisms and the relevance of this modulation in nonmammalian vertebrates are yet to be established. In zebrafish corticotrope-deficient larvae, it has been demonstrated that dexamethasone was able to rescue high-amplitude circadian cell cycle rhythms [49].

Here, we investigated the putative role of GCs as synchronizing agents of* Danio rerio *embryonic cells ZEM-2S and the evoked regulation of melanopsins, GC receptor,* per1b, *and* cry1b *expression in this photosensitive cell line.

## 2. Material and Methods

### 2.1. ZEM-2S Cell Culture

*Danio rerio *embryonic cells (ZEM-2S) (gently donated by Professor Mark Rollag, Uniformed Services University of the Health Sciences, USA, and originally purchased from ATCC, CRL-2147, Manassas, VA, USA) were kept in 50% Leibowitz L-15, 35% Dulbecco's Modified Eagle Medium (DMEM), 15% Ham's F12 medium (Athena, Campinas, Brazil), and 15 mM 4-(2-Hydroxyethyl)-1-piperazineethanesulfonic acid (HEPES) (Life Technologies, Carlsbad, CA, USA), supplemented with 10% fetal calf serum (Vitrocell, Campinas, SP, Brazil), 1% antibiotic/antimycotic solution (10,000 U penicillin/10,000 U streptomycin/25 *µ*g amphotericin B, Life Technologies, Carlsbad, CA, USA), and all-*trans-*retinal at final concentration of 10^−7^ M (Sigma, St. Louis, MO, USA), pH 7.2, at 28°C.

For the experimental protocols the serum concentration was reduced to 2%. Cells were handled in the dark under a red safelight (7 W Konex bulb and Safelight Filter GBX-2, Kodak, Rochester, NY, USA).

### 2.2. Immunocytochemistry of Opn4m1 and Opn4m2

The 15 N-terminal sequences with an appended C-terminal cysteine of* D. rerio *Opn4m1 (MSGAAHSVRKGISC) and Opn4m2 (MSHHSSWRGHHCAPGC) were conjugated to keyhole limpet hemocyanin and inoculated into rabbits to obtain the primary antisera (Covance Labs, Denver, PA, USA). The primary antisera were previously tested in a panel of dilutions ranging from 1 : 100 to 1 : 1000, and good staining was observed only at 1 : 100 and 1 : 250 for both antibodies. The antisera and the Cy3-labeled secondary anti-rabbit antibody (Jackson ImmunoResearch, West Grove, PA, USA, 1 : 500) were diluted in incubation buffer (1% bovine serum albumin, 0.25% carrageenan lambda, and 0.3% Triton X-100, all from Sigma-Aldrich, St. Louis, MO, USA).

The expressions of Opn4m1 and Opn4m2 were evaluated in ZEM-2S cells kept in DD condition for 3 days and subject to 1 hour of white light pulse (650 lux) at the beginning of the 4th day. Control cells were kept in DD for the duration of the experiment. Twenty-four hours after the light pulse, cells were fixed with 4% paraformaldehyde (Electron Microscopy Sciences, Hatfield, PA, USA) in phosphate buffered saline (PBS) at 4°C for 30 min. The blockade of nonspecific sites was made with 6% normal goat serum plus 22.52 mg/mL glycine (both from Sigma-Aldrich, St. Louis, MO, USA) in PBS at 4°C for 1 h, followed by incubation with the primary antibodies (anti-Opn4m1, 1 : 250, and anti-Opn4m2, 1 : 100) overnight at 4°C. The cells were then incubated with the secondary antibody for 1 h at room temperature, mounted with DAPI-Vectashield hard aqueous medium (Vector Laboratories, Burlingame, CA, USA) and coverslipped. Photographs were taken in an inverted fluorescence microscope Axiovert 40CFL (Zeiss, Oberkochen, Germany).

### 2.3. Image Flow Cytometry

To determine the expression of Opn4m1 and Opn4m2 proteins at light and dark phases, we quantified the fluorescence of cells using imaging flow cytometry (FlowSight, Amnis, EMD-Millipore, Seattle, WA, USA). ZEM-2S cells (2.10^6^) were kept in LD cycle for 3 days and on the 4th day the cells were fixed with 4% paraformaldehyde at ZT4 and ZT16. The material was then subject to similar procedure as described in the immunocytochemistry section, incubated in anti-Opn4m1 or anti-Opn4m2 polyclonal antibodies at 1 : 250 and 1 : 100 dilutions, respectively, followed by incubation with a sheep FITC-conjugated anti-rabbit antibody at 1 : 200 dilution (Sigma-Aldrich, St. Louis, MO, USA). Negative controls were incubated with FITC-conjugated anti-rabbit antibody, in the absence of the primary antibody. The analysis of 10,000 cell counts per sample was performed by IDEAS software (Amnis, EMD-Millipore, Seattle, WA, USA) based on scatter plot of bright field area versus aspect ratio and expressed as normalized frequency based on FITC fluorescence intensity.

### 2.4. Temporal Expression of Melanopsin and Clock Genes

ZEM-2S cells (10^6^ cells/25 cm^2^ flask) were seeded in 2% serum-supplemented medium and subject to DD or 12 : 12 LD cycle (lights-on: 9:00 h/lights-off: 21:00 h, 680 lux or 99.28 mW/cm^2^, 2.5 × 10^−14^ photons s^−1^ cm^−2^, full spectrum of white light Ecolume, 8 W cool white light bulb, model YZ8W/8 W cool white fluorescent tube, T5-8 W, and SCT) for at least 5 days, at 28°C. On the following day, total RNA was extracted every 4 h over a day. Each protocol was repeated at least twice, with two or three flasks per time point to obtain *n* = 4 to 6.

### 2.5. Dexamethasone Assays

#### 2.5.1. One DEX Pulse

ZEM-2S cells were kept in LD cycles and in the beginning of the dark phase of the 5th day the culture medium was replaced with medium containing 3 · 10^−9^ M DEX. Twelve hours later the culture medium was replaced by fresh medium, and on the 6th day total RNA was extracted every 4 h during 24 h.

#### 2.5.2. Three DEX Pulses

ZEM-2S cells were kept in LD cycles and treated with three 12-hour pulses of 3 · 10^−9^ M DEX during the dark phase of the 3rd, 4th, and 5th days. After each 12-hour pulse the DEX containing medium was replaced with fresh medium. Twelve hours after the third DEX treatment, the culture medium was replaced by fresh medium, and on the 6th day total RNA was extracted every 4 h during 24 h.

#### 2.5.3. Five DEX Pulses

ZEM-2S cells were kept in DD conditions and were treated with 2-hour pulses of 10^−7^ M DEX for 5 days starting at ZT 0. After each 2-hour pulse the DEX containing medium was replaced with fresh medium. On the 6th day total RNA was extracted every 4 h during 24 h.

The medium of the control cells was changed simultaneously with DEX-treated cells, totaling up to 2, 6, or 10 medium changes, respectively. DEX concentration and application time were based on Gilchrest and colleagues [48], who reported that plasma cortisol of rainbow trout* (Oncorhynchus mykiss)* peaks in the dark phase.

### 2.6. Total RNA Extraction, Reverse Transcriptase-Polymerase Chain Reaction (RT-PCR), and Quantitative PCR (qPCR)

Total RNA was extracted with TRIzol Reagent (Ambion, Foster City, CA, USA) according to the manufacturer's instructions. The RNA* pellet* was resuspended in DEPC H_2_O (diethylpyrocarbonate, Ambion, Foster City, CA, USA) and treated with DNAse according to the manufacturer's protocol (turbo DNA-Free™, Ambion, Foster City, CA, USA). RNA concentration (OD260) was determined in a spectrophotometer (Nanodrop, Wilmington, DE, USA), and 2 *µ*g was submitted to RT-PCR (SuperScript III, Invitrogen, Carlsbad, CA, USA) using random primers. The reaction protocol was as follows: 5 min at 65°C and 1 min at 4°C; after the enzyme addition, 5 min at 25°C and 50 min at 50°C; and 15 min at 70°C in a thermocycler (Eppendorf, Hauppauge, NY, USA).

Using TaqMan approach to simultaneously analyze the expression of* per1b*,* cry1b,* and ribosomal 18S RNA or* opn4m1*,* gr*, and 18S RNA we prepared a mix of primers (300 *η*M for genes and 50 *η*M for 18S RNA, Table 1), fluorescent probes (200 *η*M for genes and 50 *η*M for 18S RNA, Table 1), and Supermix 2X (Bio-Rad Laboratories, Hercules, CA, USA, or Life Technologies, Carlsbad, CA, USA), supplemented to final concentrations of 400 *µ*M dNTPS, 6 mM MgCl_2_, and 0,1 U/*µ*L Platinum Taq DNA polymerase (Life Technologies, SP, Brazil). Each experimental cDNA was run in triplicate in 96-well plates.

For* opn4m2 *and 18S RNA, the qPCR reactions contained iQ™ SYBR® Green Supermix 2X (Bio-Rad Laboratories, Hercules, CA, USA) or SYBR® GreenER™ qPCR SuperMix for iCycler® 2X (Life Technologies, Carlsbad, CA, USA) and specific primers, in final concentrations of 300 *η*M for* opn4m2 *and 50 *η*M for 18S RNA, respectively, in independent solutions. Primers and probes for* Danio rerio* were designed based on sequences obtained from GenBank (http://www.ncbi.nlm.nih.gov/Genbank) and synthesized by IDT (Coralville, IA, USA). 18S RNA was used to normalize the values of specific genes, since it did not vary among time points under the various experimental conditions and has a stable expression in many tissues of zebrafish [50].

Primers' efficiency was determined using serial dilutions (1, 1 : 2, 1 : 4, 1 : 8, and 1 : 16) of a single cDNA sample. The triplicate mean CT of each gene was plotted in the *y*-axis and the log of cDNA dilutions in the *x*-axis. The efficiency for each primer pair was calculated according the equation: 10^∧^(−1/*x*) − 1*∗*100, in which *x* corresponds to the inclination angle of the linear regression curve. Values between 90% and 110% were considered as indicators of appropriate efficiency.

All assays were performed in iCycler or iQ5 (Bio-Rad Laboratories, Hercules, CA, USA) thermocycler, with the following protocols: multiplex, 1 cycle of 7 min at 95°C followed by 45 cycles of 30 sec at 95°C and 30 sec at 60°C; SYBR Green, 2 min at 50°C, 8:30 min at 95°C, 45 cycles of 15 sec at 95°C, 1 min at 60°C, 1 min at 95°C, 1 min at 55°C, and 80 cycles of 10 sec at 55°C with a gradual increase of 0.5°C. Negative controls without templates were routinely included.

### 2.7. Statistical Analysis

The ΔΔCT method was used to analyze relative changes in gene expression by qPCR. The CT was obtained comparing the number of cycles in control and experimental wells and among the various time points, by passing a threshold line through the geometric portions of the amplification curves. The ΔCT was calculated as the difference between the CT for 18S RNA and that for the gene of interest; the smallest value obtained in the control group or in a time point was then subtracted from each ΔCT, originating the ΔΔCT. This value was then used as the negative exponential of base 2. To assess temporal variation among different time points (ZTs), means were compared by one way analysis of variance (ANOVA) followed by Tukey test; to compare control and experimental groups along time, two way ANOVA followed by Bonferroni test was used. All data were analyzed and graphed by GraphPad Prism 5 software, and the differences were considered statistically significant when *p* ≤ 0.05.

## 3. Results and Discussion 

### 3.1. Melanopsins and Glucocorticoid Receptor

The striking diversity of extra genes expressed in* D. rerio *explained by genome duplication event in the teleost lineage may reflect either redundant or specialized functions performed by the multiple copies [19]. However, the fact that the five melanopsin genes are differentially expressed in many neuronal cell types in the adult fish retina leads toward the presence of a finely tuned/sophisticated melanopsin system [29, 50].

By contrast, not all* opn4 *mRNAs were detected in earlier stages of development, as demonstrated in* D. rerio *ZEM-2S cells, where* opn4m1 *and* opn4m2 *genes are more expressed as compared to* opn4m3*,* opn4x1*, and* opn4x2 *genes, whose expression was negligible [14]. Thereby, in this study only* opn4m1 *and* opn4m2 *genes were analyzed besides the glucocorticoid receptor (*gr*) gene which was also found to be expressed in this cell line.

**Figure 1:**
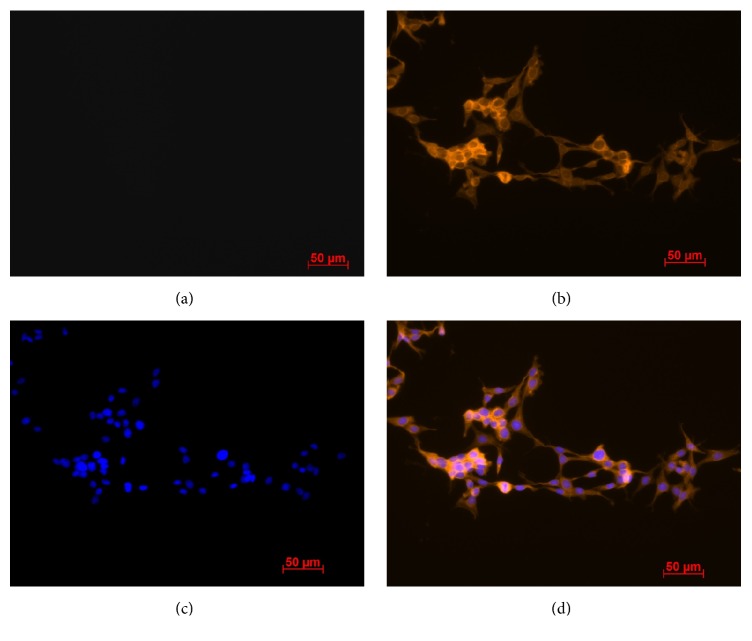
Evidence of Opn4m1 immunoreactivity in* D. rerio *ZEM-2S cells. (a) Control preparation in which the primary antibody was omitted; (b) Opn4m1 labeling revealed by Cy3-secondary antibody in orange showing the protein localization in the cell membrane; (c) DAPI-labeled nuclei in blue; (d) merged (b) and (c) images. Photomicrographies were taken with Axiocam MRm camera (Zeiss) and pseudocolored with Axiovision Software (Zeiss). Scale bar 50 *µ*m (200x magnification).

**Figure 2:**
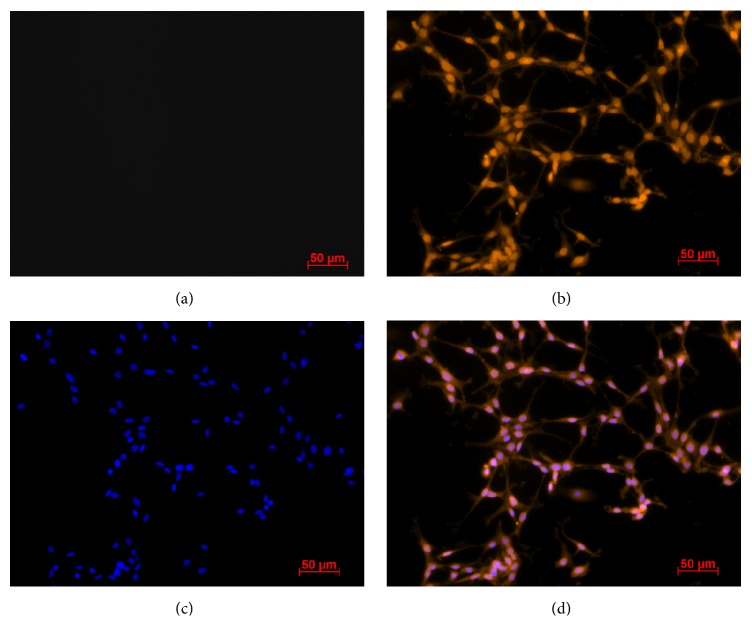
Evidence of Opn4m2 immunoreactivity in* D. rerio *ZEM-2S cells. (a) Control preparation in which the primary antibody was omitted; (b) Opn4m2 labeling revealed by Cy3- secondary antibody in orange showing nuclear localization of the protein; (c) DAPI-labeled nuclei in blue; (d) merged (b) and (c) images. Photomicrographies were taken with Axiocam MRm camera (Zeiss) and pseudocolored with Axiovision Software (Zeiss). Scale bar 50 *µ*m (200x magnification).

**Figure 3:**
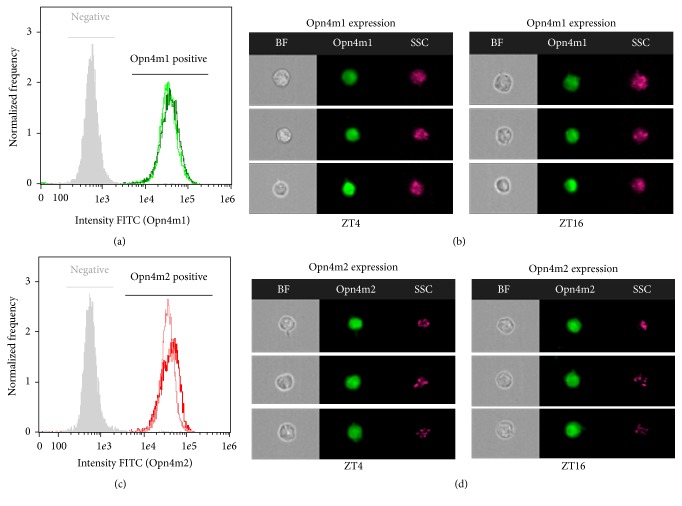
Imaging flow cytometry of ZEM-2S cells stained for Opn4m1 and Opn4m2. Note the displacement of fluorescence intensity to the right in comparison to the isotype control (negative) after cells were incubated with anti-Opn4m1 and ant-Opn4m2 antibodies, which demonstrates that ZEM-2S cells express both proteins. (a) Frequency histograms of fluorescence intensity for negative control or Opn4m1-positive cells obtained at ZT4 (light-green line, *n* = 4 flasks) and ZT16 (dark-green line, *n* = 4 flasks); (b) representative images of bright field, Opn4m1-FITC fluorescence (green), and side scatter (SSC) of cells expressing Opn4m1 at ZT4 (left lane) or ZT16 (right lane); (c) frequency histograms of fluorescence intensity for negative control or Opn4m2-positive cells obtained at ZT4 (light-red line, *n* = 3 flasks) and ZT16 (dark-red line, *n* = 4 flasks); (d) representative images of bright field, Opn4m2-FITC fluorescence (green) and side scatter (SSC) of cells expressing Opn4m2 at ZT4 (left lane) or ZT16 (right lane).

Here we demonstrate for the first time the presence and localization of the proteins Opn4m1 and Opn4m2 in* D. rerio *ZEM-2S cells using immunocytochemistry and imaging flow cytometry. Positive labeling for Opn4m1 (Figures 1(b) and 1(d)) was remarkably detected in the cell membrane at 1 : 250 dilution in cells kept in DD condition; no immunostaining was observed after incubation with rabbit preserum instead of the anti-Opn4m1 antiserum (Figure 1(a)). Interestingly Opn4m2 labeling is nuclear and much less evident in the membrane than Opn4m1 (Figures 2(b) and 2(d)). Control preparation incubated with preserum showed no immunolabeling (Figure 2(a)). Although it is commonly accepted that opsins are typically located in the cell membrane, similar results were also reported in* Xenopus laevis *melanophores [16]. These cells express two melanopsins,* Opn4x *and* Opn4m*, which are also differentially distributed in the cell: immunoreactivity for* Opn4x *was seen in the cytoplasm and cell membrane whereas* Opn4m *is strongly expressed in the nucleus [16]. The precise explanation for this distinct distribution is elusive and further investigation is needed to solve this question. However, a recent study using mammalian cells brought a possible explanation [51]. Mammals express only one melanopsin [25], and in melanocytes this opsin is located in the nucleus [51]. Interestingly after a 15 min white light pulse this opsin translocates to the cell membrane. In contrast, in ZEM-2S cells, light pulse was not able to induce the translocation of Opn4m2 to membrane (see Supplemental Figure 1 in Supplementary Material available online at https://doi.org/10.1155/2017/8459385). The differential response between nonmammalian and mammalian melanopsins may be due to the fact that mammals express only one melanopsin gene/protein, while* D. rerio* and* X. laevis* express multiple melanopsins. In* D. rerio* Opn4m1 found in the cell membrane likely works as a photosensor, while the nuclear melanopsin (Opn4m2) could function as a transcription factor. In addition, analyses of flow cytometry imaging revealed that the majority of the cells were positive for Opn4m1 (ZT4 91.9%  ± 7.5% versus ZT16 99.07%  ± 0.12%) and Opn4m2 (ZT4 99.75%  ± 0.1% versus ZT16 99.43%  ± 0.20%). However no difference in fluorescence intensity between ZTs was found (Figure 3). Taken together, these data suggest that although* D. rerio* expresses more than one melanopsin protein and there was no variation between light and dark phases, only Opn4m1, located in the membrane, may be responsible for transducing light stimulus.

**Figure 4:**
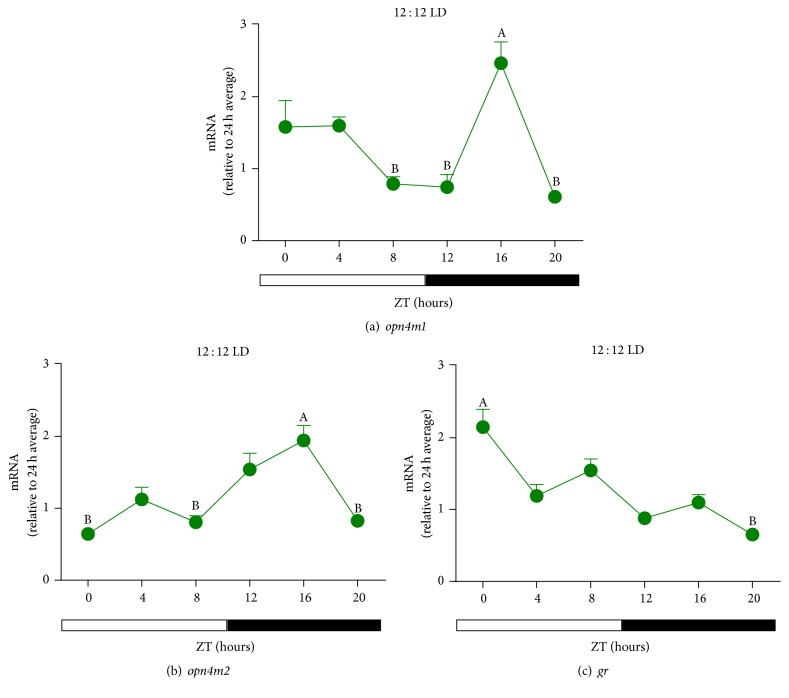
Time course of (a)* opn4m1*; (b)* opn4m2*; (c)* GR* gene expression in* D. rerio* ZEM-2S cells exposed to LD (12 : 12) cycles. Each value is the mean (*n* = 4–6 flasks), ±SEM, of transcripts normalized by ribosomal 18S RNA and expressed relative to 24 h average of each gene. A significantly different from B (*p* < 0.05, one way ANOVA, followed by Tukey).

LD cycle is a well-known* zeitgeber *that synchronize the clock components both in vitro and in vivo, allowing the rhythmic expression of several clock-controlled molecules. In this context, we analyzed the temporal profile of melanopsins and* gr* gene expression in LD cycles. ZEM-2S cells kept in LD cycles showed similar temporal variation of* opn4m1 *and* opn4m2 *genes which peaked in the dark phase at ZT16 (Figures 4(a) and 4(b)). Although* opn4m1* transcript peaked at ZT16 (Figure 4(a)), no difference of protein expression was seen between ZTs (Figure 3), which could be happening in other ZTs. As to* gr*, its expression slightly decreased over the course of the 24 h; that is, higher* gr* expression was seen at ZT0 in comparison to ZT20 (Figure 4(c)).

**Figure 5:**
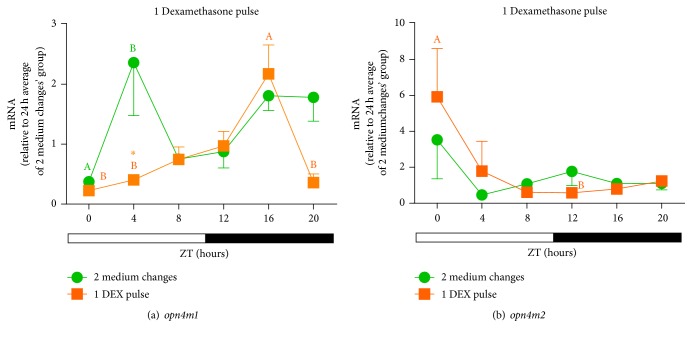
Time course of* opn4m1* (a) and* opn4m2 *(b) gene expression in* D. rerio* ZEM-2S cells exposed to LD (12 : 12) cycles and subject to 2 medium changes (control) or 1 DEX pulse. Each value is the mean (*n* = 4–6 flasks), ±SEM, of transcripts normalized by ribosomal 18S RNA and expressed relative to 24 h average of each gene after 2 medium changes. A significantly different from B demonstrated by one way ANOVA, followed by Tukey (*p* < 0.05). Asterisk means significantly different from the respective control at the same time point (*p* < 0.05, two way ANOVA followed by Bonferroni).

**Figure 6:**
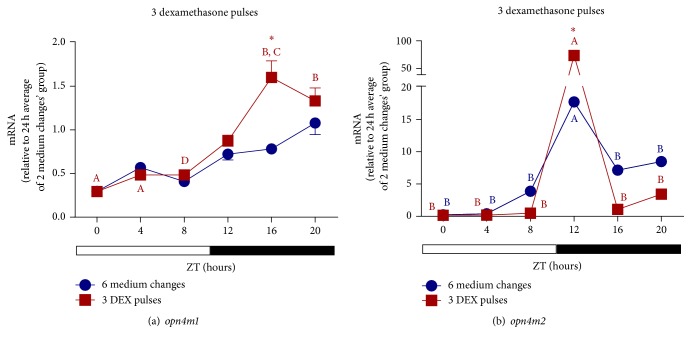
Time course of* opn4m1* (a) and* opn4m2 *(b) gene expression in* D. rerio* ZEM-2S cells exposed to LD (12 : 12) cycles and subject to 6 medium changes (control) or 3 DEX pulses. Each value is the mean (*n* = 4–6 flasks), ±SEM, of transcripts normalized by ribosomal 18S RNA and expressed relative to 24 h average of each gene after 2 medium changes. A significantly different from B and C from D demonstrated by one way ANOVA, followed by Tukey (*p* < 0.05). Asterisk means significantly different from the respective control at the same time point (*p* < 0.05, two way ANOVA followed by Bonferroni).

The oscillatory profiles of both melanopsins in LD cycle (Figures 5(a) and 5(b)) were altered after 2 medium changes (control cells), the peak shifting to the light phase. Nevertheless, one DEX pulse was able to shift* opn4m1* peak of expression back to the dark phase (Figure 5(a)), but not of* opn4m2 *(Figure 5(b)). Surprisingly, 6 medium changes (control cells for 3 hormone pulses) abolished the temporal oscillation of* opn4m1 *seen in LD before medium changes, and 3 pulses of DEX restored its temporal variation, defining a robust circadian rhythm, which peaked again during the dark phase (Figure 6(a)). For* opn4m2*, similar medium changes did not affect the oscillation seen in LD without manipulation (peak in the dark), and 3 pulses of DEX remarkably increased mRNA levels keeping the peak in the beginning of the dark phase (Figure 6(b)). These results demonstrate that glucocorticoids restored* opn4m1 *temporal profile in LD, which had been affected by medium changes, indicating that this opsin is more susceptible to dexamethasone treatment than* opn4m2*. Despite the fact that the glucocorticoid responsive element (GRE) has yet to be demonstrated in the melanopsin genes, its presence in* opn4m1 *but not in* opn4m2 *promoter might explain the differential response of these melanopsins to glucocorticoids.

Therefore, it is most likely that a functional circadian photopigment is present in these embryonic cells associated with the adjustment of the clock molecular machinery to light conditions. It was once thought that* cry *genes could exert a role in photoentrainment of vertebrate biological clocks because they mediate a variety of light responses, and their evolutionary history is related to photolyases [52]. In particular,* cry1b *and* cry3 *were indicated as potential candidates for this function in* D. rerio *Z3 cells [34]. More recently, however, we have demonstrated through pharmacological intervention in the rhabdomeric signaling pathways that melanopsins are the strongest candidate to exert the photopigment role in ZEM-2S cells [14]. Altogether, these results and the literature data strengthen the possibility that this opsin is the functional photopigment in* D. rerio *ZEM-2S cells.

**Figure 7:**
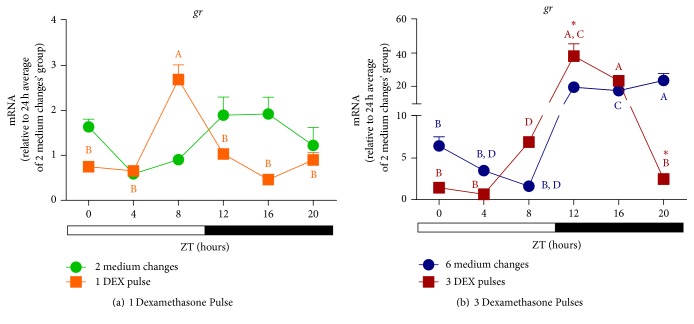
Time course of* gr* gene expression of* D. rerio* ZEM-2S cells exposed to LD (12 : 12) cycles and subject to (a) 2 medium changes (control) or 1 DEX pulse; (b) 6 medium changes (control) or 3 DEX pulses. Each value is the mean (*n* = 4–6 flasks), ±SEM, of transcripts normalized by ribosomal 18S RNA and expressed relative to 24 h average of* gr* after 2 medium changes. A significantly different from B and C from D demonstrated by one way ANOVA, followed by Tukey (*p* < 0.05). Asterisk means significantly different from the respective control at the same time point (*p* < 0.05, two way ANOVA followed by Bonferroni).

The main mechanism of Gr regulation is performed by homologous downregulation, which occurs by decreasing the rate of* gr* transcription [53]. Our results confirm that dexamethasone affects the expression of its own receptor along 24 hours. One DEX pulse imposed a temporal variation with a peak of* gr *mRNA in the light phase (Figure 7(a)). Six medium changes or 3 pulses of DEX drastically increased the level of* gr *transcripts, respectively, around 50- and 80-fold, the values seen after 2 medium changes, and shifted the peak to the light-dark transition (Figure 7(b)).

In mammals, it is known that basal levels of glucocorticoid hormones exhibit marked circadian variation produced by secretory episodes with constant frequency and variable amplitude, which would exert an impact on the pattern of target gene transcription via GREs in target gene promoters [54]. Furthermore, nongenomic pathways have been described to be triggered by membrane-associated GR and second messengers [55–60].

Indeed, since the last decade this hormone class has been related to circadian regulation of peripheral clocks [45, 47, 48, 61–63]. The influence of DEX pulses on* opn4m1 *and* opn4m2 *expression shows that there may be a direct or indirect effect involving a signaling pathway evoked by Gr-glucocorticoid binding.

### 3.2. Clock Genes

The presence of multiple clock genes expressed in embryos and larvae of* D. rerio* and the ability to respond to light support the embryonic cell line ZEM-2S as a valuable model to study clock regulation. Although embryonic clocks are differentially regulated from those in the adult organism [64], photic signals and, consequently, an active phototransduction system, during blastula to early segmentation stages, are indispensable to maturation of a functional circadian clock [40].

Despite the relevance of peripheral clocks for understanding the organization of circadian system of an organism, the regulation of peripheral timing remains to be elucidated [65]. Hormones, whose temporal production and secretion are certainly defined by the master biological clock, have been particularly investigated as synchronizing agents of mammalian peripheral clocks [42, 66]. For nonmammalian vertebrates, the hormonal action on peripheral clocks has been less studied and its investigation could uncover evolutionary features of this regulation.

Circadian expression of clock genes in the presence of glucocorticoids has been evaluated in mammalian models of peripheral clocks. Balsalobre and coworkers [61] showed that 1-hour DEX-treatment was able to increase* Per1 *mRNA levels and promote a rhythmic expression of* Per1*,* Per2*,* Per3*, and* Cry1 *and clock-controlled genes* Rev-erbα*and* Dbp *in rat-1 fibroblasts. Dexamethasone downregulated* Rev-erbα*transcripts in rat primary hepatocytes [48], and in cultured mouse liver cells, chronic prednisolone treatment induced* Per1 *mRNA expression and attenuated oscillatory profiles of* Per2*,* Rev-erbα, *and* Bmal1 *[62]. All* PER* and* BMAL1 *genes were also affected by glucocorticoids in human peripheral blood mononuclear cells [63].

**Figure 8:**
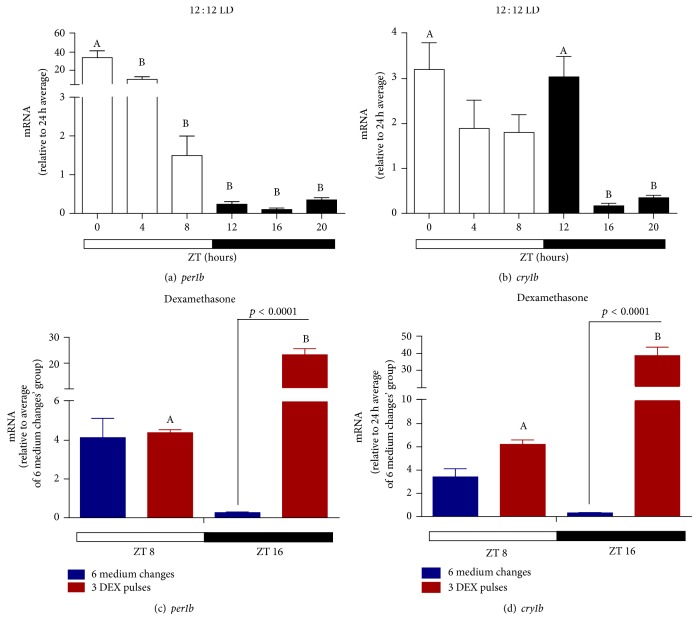
Time course of* per1b and cry1b* expression in* D. rerio* ZEM-2S cells exposed to LD (12 : 12) cycles. (a)* per1b* and (b)* cry1b* in nonmanipulated cells; (c)* per1b* in cells subject to 6 medium changes (blue bars) or 3 DEX pulses (red bars); (d)* cry1b *in cells subject to 6 medium changes (blue bars) or 3 DEX pulses (red bars). Each value is the mean (*n* = 4–6 flasks), ±SEM, of transcripts normalized by ribosomal 18S RNA. (a) and (b) are expressed relative to 24 h average. (c) and (d) relative to 24 h average of 6 medium changes. Temporal variation: A is different from B (*p* < 0.05, one way ANOVA, followed by Tukey posttest), and DEX-treated group is different from the respective control (*p* < 0.0001, two way ANOVA, followed by Bonferroni posttest).

**Figure 9:**
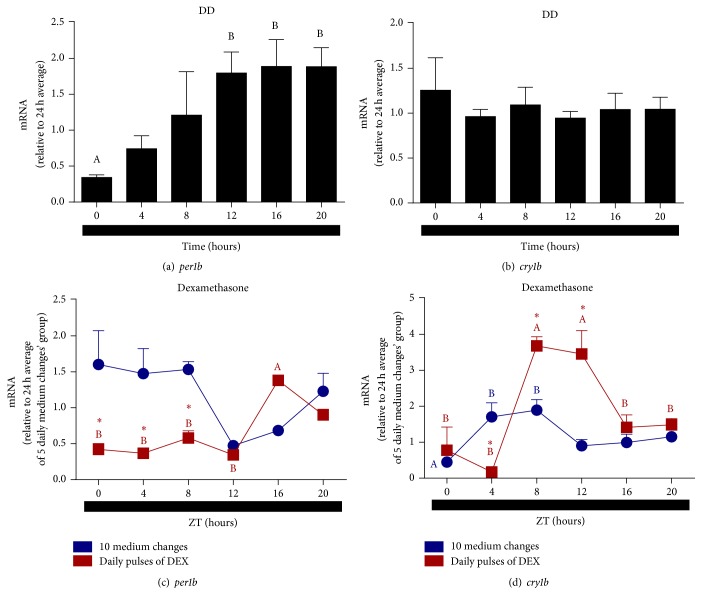
Time course of* per1b* and* cry1b* expression in* D. rerio* ZEM-2S cells subject to DD. (a)* per1b* and (b)* cry1b* in nonmanipulated cells; (c)* per1b* in cells subject to 10 medium changes (blue symbols) or 5 DEX pulses (red symbols); (d)* cry1b *in cells subject to 10 medium changes (blue symbols) or 5 DEX pulses (red symbols). Each value is the mean (*n* = 4–6 flasks), ±SEM, of transcripts normalized by ribosomal 18S RNA. (a) and (b) are expressed relative to 24 h average, (c) and (d) relative to 24 h average of 6 medium changes. A significantly different from B demonstrated by one way ANOVA, followed by Tukey (*p* < 0.05). Asterisk means significantly different from the respective control at the same time point (*p* < 0.05, two way ANOVA followed by Bonferroni).

Having this in mind we investigated the temporal expression profile of clock genes* per1b *and* cry1b *in the ZEM-2S cells in response to LD cycles and DEX treatment. Under LD cycles, mRNA levels of* per1b *presented a pronounced circadian oscillation with a remarkable 40-fold difference between light and dark phase transcripts (Figure 8(a)), peaking at ZT0 and gradually decreasing till the dark phase. For* cry1b*, in turn, a discrete temporal variation was observed with higher points of expression at the transitions of light-dark and dark-light phases (Figure 8(b)). Thus, comparing to the expression of* opn4m1 *and* opn4m2 *(Figures 4(a) and 4(b)), we found that the expression of* per1b *(Figure 8(a)) is in antiphase with melanopsin expression while no association was found for* cry1b *expression (Figure 9(b)).

Understanding the peripheral clock regulation in vivo is a complicated task, since several hormones may act to set the clock machinery. Interesting in nonmammalian cells that are able to directly detect light, hormones seem to play a minor role in the synchronization of clock components [15, 17]. Our results demonstrate a remarkable effect of dexamethasone pulses on clock genes. At ZT16 (dark phase) the expression of* per1b *(Figure 8(c)) and* cry1b *(Figure 8(d)) increased 30- and 50-fold, respectively, after 3 DEX pulses. This increase of* per1b *and* cry1b *in the dark phase coincides with a high expression of* opn4m1 *also after 3 DEX pulses.

The rhythmic profile of clock genes is clearly elicited by LD cycles since, in the absence of light,* per1b *exhibits a very slight variation, with a low amplitude peak during the subjective night (Figure 9(a)), while* cry1b* is constitutively expressed (Figure 9(b)). In a previous study, we have shown that medium change in DD induces a similar profile to LD, but with much lower amplitude [11]. A rhythm of lower amplitude has been shown for clock genes in zebrafish embryonic cell lines as PAC-2 (*clock, *[20]) and in Z3 cells (*clock*,* per1*,* per3*,* bmal1,* and* bmal2, *[39]), as well as in cultured zebrafish heart and kidney (*clock *[20]) maintained under constant conditions. The direct responsiveness to light stimulus is certainly the most remarkable characteristic of zebrafish cells. Similarly to* per1b *expression profile in ZEM-2S cells,* per1 *and* per3 *are rhythmically expressed in Z3 cells under LD cycles [39]. Phylogenetic studies revealed that the four* per *genes,* per1a*,* per1b*,* per2 *and* per3, *of* D. rerio *are, apparently, differentially regulated, with distinct spatial and temporal patterns [38]. In particular,* per2 *is light-inducible and does not oscillate in constant conditions [14, 18, 19].

We also investigated the ability of DEX to synchronize clock gene expression in the absence of light. Although* per1b* expression in cells subject to medium changes in DD seems to oscillate with higher values at ZTs 0, 4, and 8, no statistical differences were found (Figure 9(a)). DEX treatment in DD promoted a temporal oscillation of* per1b* transcription with high expression at ZT16 in comparison to ZTs 0, 4, 8, and 12. In addition, DEX induced a reduction of* per1b *expression at ZTs 0, 4, and 8, as compared to the untreated group. DEX treatment in DD (Figure 9(a)) did mimic LD cycle effects (Figure 8(a)) on* per1b* expression, inducing an oscillation, but with different profile. That is, in the first condition it peaked at ZT16 whereas in LD it was higher at ZT0. If we compare the effects of DEX in DD and in LD, new information arises from the results: in DD DEX decreased (Figure 9(a)) while in LD it increased (Figure 8(c))* per1b* expression. On the other hand, cells kept in DD subject to medium changes showed a temporal variation of* cry1b*, with higher expression at ZTs 4 and 8 in comparison to ZT0. After DEX treatment we observed a shift in the peak expression of* cry1b* (Figure 9(b)) from ZTs 4 and 8 (control cells, medium changes) to ZTs 8 and 12. Unlike* per1b*,* cry1b* transcription was increased by DEX in both DD (Figure 9(b)) and LD (Figure 8(d)).

Glucocorticoids exhibit a strong daily rhythm in the plasma of many vertebrates from fish to mammals [67–69]. This rhythmic glucocorticoid release is a signal to peripheral oscillators in mammalian liver, heart, lung, stomach, and kidney [46, 62]. However, the role of glucocorticoids in the regulation of clock gene expression in fish is still speculative. Sánchez-Bretaño and colleagues demonstrated, in a very elegant work in* Carassius auratus*, that, similarly to what we found in* D. rerio *cultured embryonic cells, DEX induces* per *genes in the liver in in vivo assays as well as in cultured hepatocytes [70]. In light responsive peripheral clocks, light has been demonstrated to prevail in regulating opsins and clock genes, as compared to many hormones, such as melatonin, *α*-MSH [13], and endothelin [15]. Despite the fact that* D. rerio *ZEM-2S cells are also photosensitive, glucocorticoids seem to be as relevant as light to regulate photoreception and the circadian system.

## 4. Conclusions

In summary, we report here, for the first time, the localization and daily variation of the melanopsin proteins, Opn4m1 and Opn4m2, in a nonmammalian photosensitive clock, the* D. rerio *ZEM-2S embryonic cells. It is interesting to mention that unlike mammals in which the single melanopsin migrates from the nucleus to the cell membrane in response to light, in ZEM-2S cells, the nuclear melanopsin does not relocate after light stimulus. We provide evidences of DEX stimulatory effect on all genes, except for* per1b* in ZEM-2S cells kept under DD. These results demonstrate the remarkable influence of glucocorticoids in all organizational levels of peripheral* D. rerio* circadian system, that is, photoperception, clock core, and clock-controlled genes such as* gr*.

## Supplementary Material

Danio rerio possess five melanopsin genes (Davis et al., 2011); among them, opn4m1 and opn4m2 were reported as the most expressed melanopsins in ZEM-2S cells (Ramos et al., 2014). The cellular compartments where Opn4m1 and Opn4m2 are expressed were identified here by immunocytochemistry. Surprisingly Opn4m2 was found in the nucleus when ZEM-2S cells were kept in DD conditions. So, we investigated the effects of white light pulse in promoting melanopsin translocation. Danio rerio ZEM-2S cells were kept for 3 days in DD condition and subject to 1 hour of white light pulse (650 lux) in the beginning of the 4th day. The incubation of ZEM-2S cells with anti-Opn4m2 at 1 : 100 dilution demonstrated that white light pulse did not altered Opn4m2 nuclear location. On the other hand, in mammalian melanoma cells, a short white light pulse (15 min) promotes melanopsin translocation from the nucleus to the cell membrane (de Assis et al., 2016). Davies, W. I. L. et al. (2011). Functional diversity of melanopsins and their global expression in the teleost retina. Cell. Mol. Life Sci. 68(24), 4115–4132. Ramos, B. C. R., Moraes, M. N. C. M., Poletini, M. O., Lima, L. H. R. G., and Castrucci, A. M. L. (2014) From blue light to clock genes in zebrafish ZEM-2S cells. PLoS ONE 9 (9), Article ID e106252. de Assis, L. V. M., Moraes, M. N., da Silveira Cruz-Machado, S., and Castrucci, A. M. L. (2016) The effect of white light on normal and malignant murine melanocytes: a link between opsins, clock genes, and melanogenesis. Biochim. Biophys. Acta 1863(6), 1119–1133.

## Figures and Tables

**Table 1:** Primers and probes for qPCR assays.

*Templates* (access number)	*Primers and probes*	*Final concentration*
18S RNA X03205.1	Forward: 5′-CGGCTACCACATCCAAGGAA-3′	50 nM
	Reverse: 5′-GCTGGAATTACCGCGGCT-3′	50 nM
	Probe: 5′TexRd/TGCTGGCACCAGACTTGCCCTC/BHQ_2/-3′	50 nM

*opn4m1* GQ925715.1	Forward: 5′-GGGCAACTTCCTGGTCATCTATG-3′	300 nM
	Reverse: 5′- AGGGAACTGACACTGGAACCA-3′	300 nM
	Probe: 5′-/5Cy5/AGGAGCCGGACCCTGAGGACCC/BHQ_2/-3′	200 nM

*opn4m2* AY078161	Forward: 5′-GCGATTGTCTTCTGCCTCTGA-3′	300 nM
	Reverse: 5′-AGGGAACTGACACTGGAACCA-3′	300 nM
	Probe: 5′-/6FAM/AGTGATTCTTGCTGGACTGAGAGTGAGGCT/BHQ_1/-3′	200 nM

*gr* NM_001020711.2	Forward: 5′-GAGGAGAACTCCAGCCAGAAC-3′	300 nM
	Reverse: 5′-TTCACAAAGGTGTAGAAGCAGAAG-3′	300 nM
	Probe: 5′-/5HEX/AGGAGTCCACCCACCAAGTCGTGC/BHQ_1/-3′	200 nM

*per1b* NM_212439.2	Forward: 5′-AGCTCAAACTCTCACAGCCCTT-3′	300 nM
	Reverse: 5′-TCAGAGCTGGCACTCAACAGA-3′	300 nM
	Probe: 5′-/5Cy5/TCCACCCAGCAGTTCTCTGGCATACA/BHQ_2/-3′	200 nM

*cry1b* NM_131790.4	Forward: 5′-CGTCTCTGGAGGAGCTCGG-3′	300 nM
	Reverse: 5′-TCTCCCCCGGGCCAC-3′	300 nM
	Probe: 5′-/5HEX/TTTGAAACAGAGGGACTGTCCACTGCTG/BHQ_1/ 3′	200 nM

FAM = carboxyfluorescein; TexRd = Texas Red fluorophore; Cy5 = Cyanine 5 fluorophore; Hex = 6-carboxy-2,4,4,4,7,7-hexachlorofluorescein succinimidyl ester fluorophore; BHQ_1 = black hole quencher 1; BHQ_2 = black hole quencher 2.

Gene nomenclature according to http://www.genenames.org for *Homo sapiens*; http://www.informatics.jax.org/genes.shtml for *Mus musculus*; https://wiki.zfin.org for *Danio rerio*.
